# Dual Micromechanical Interlocking Through Filler Surface Modification for Enhanced Dental Composites

**DOI:** 10.3390/polym17172384

**Published:** 2025-08-31

**Authors:** Hongyan Chen, Jiaxuan Lyu, Jia Nie, Xuhui Wang, Na Yang, Sheng Han, Mingliang Zhou

**Affiliations:** 1 School of Chemical and Environmental Engineering, Shanghai Institute of Technology, Shanghai 201418, China; chenhy@sit.edu.cn (H.C.); 2 Department of Prosthodontics, Department of General Dentistry, Shanghai Ninth People’s Hospital, Shanghai Jiao Tong University School of Medicine, National Clinical Research Center for Oral Diseases, Shanghai Key Laboratory of Stomatology, Shanghai Research Institute of Stomatology, Research Unit of Oral and Maxillofacial Regenerative Medicine, Chinese Academy of Medical Sciences, Shanghai 200011, China; 3 Shanghai Stomatological Hospital, Fudan University, Shanghai 201102, China

**Keywords:** porous silica, 3D urchin-like hydroxyapatite, interface interaction, dual micromechanical interlocking, dental resin composites

## Abstract

A novel structure–functional-integrated particle featuring dual micromechanical interlocking property with resin matrix was constructed through surface modification of urchin-like serried hydroxyapatite (UHA) in this work, and the effect of this modification strategy on physicochemical and biological properties of dental resin composite was also investigated. A porous silica coating layer was anchored onto UHA surface via a simple template method in an oil−water biphase reaction system, and the coating time had a prominent effect on the coating thickness and morphology-structure of the particle. When these particles with different porous silica coating thickness were used as fillers for dental resin composite, results showed that UHA/PS5 (porous silica coating reaction time: 5 h) exhibited the optimal 3D urchin-like structure and a desirable porous silica coating thickness. Additionally, UHA/PS5 formed the best dual physical micromechanical interlocking structure when mixing with resin matrix, making the dental resin composites presented the desirable matrix/filler interfacial bonding, and the excellent physicochemical–biological properties, especially for flexural strength and water sorption-solubility. In vitro remineralization and cellular biological properties confirmed that the coating layer did not compromise their remineralization activity. The use of UHA/PSx provides a promising approach to develop strong, durable, and biocompatible DRCs.

## 1. Introduction

Dental resin composites (DRCs) are prevalent restorative materials for repairing decayed teeth since 1960s due to their superior properties [1,2,3]. These composites mainly consist of fillers and the resin matrix [4,5], where the organic resin can be polymerized to form a continuous resin matrix phase and the inorganic fillers are dispersed in the resin matrix to endow DRCs mechanical properties, wear-resisting property, and biological activity, etc. [6,7,8]. For the DRCs, the interface interaction between filler and resin matrix plays an important role in the comprehensive physicochemical–biological properties and the lifespan of DRCs [9,10], which should be capable of the transfer of the external force from the resin matrix to fillers [11,12]. Therefore, how to enhance the filler–resin matrix interface interaction is of vital importance for DRCs.

The most commonly used methods to enhance filler–resin interfacial interactions modify the surface structure and chemical properties of fillers, as filler loading in commonly used DRCs exceeds 60 wt% [13,14,15]. The chemical modification method is mainly achieved by grafting organo-functional groups onto a filler’s surface [16]. This process is complicated and the formed covalent bond between fillers and resin may be easily degraded in the humid oral environment [17,18], making the fillers de-bond from the resin matrix, thus weakening the properties of corresponding DRCs. By contrast, the physical interlocking between filler and resin matrix is a more effective way to create durable interfacial binding [19,20,21,22,23,24], which is mainly achieved by adjusting the filler’s surface morphology structure. Based on our previous research, particles with porous or three-dimensional whisker structures are suitable fillers in DRCs. Their structures enable the resin to infiltrate the pores, or allow whiskers to insert into the resin matrix under pressure, thereby forming a micromechanical interlocking interface between filler and resin matrix through a physical process.

Hydroxyapatite (Ca_10_(PO_4_)_6_(OH)_2_, HA) is a typical bioactive material, which has been introduced into DRCs due to it is the main component of hard dental tissues [25,26,27]. Our previous studies demonstrated that HA-filled DRCs showing good remineralization properties, which is important in reducing secondary caries [5,25]. Additionally, HA with urchin-like serried structure (UHA) exhibited the best reinforcing effect in DRCs than particle, whisker, or nanofiber, which mainly attributed to its unique structure. The 1D whisker of UHA plays the reinforcing roles of crack deflection and whisker pullout [28,29], and its 3D whisker structure can improve the filler–resin matrix interfacial interaction based on the physical micromechanical interlocking [30,31,32]. However, the surface of the synthesized UHA contains a large number of hydroxyl groups, which makes them prone to aggregation and water absorption [33,34]. Eventually, this reduces the mechanical properties of the DRCs in the oral environment and weakens their usability. A remedy to this problem is the surface modification of UHA, which process can occupy its hydroxyl groups with other hydrophobic chemical element [25,34] or coating layer.

Porous silica has been proven to have excellent reinforcing effects in DRCs due to its porous structure and high surface area [23,35,36]. Based on our previous studies, porous silica with appropriate pore size can effectively enhance the physical interlocking effect when mixing with resin matrix in DRCs, therefore improve the mechanical properties [37]. However, spherical porous silica has a radiating pore structure, where the pore size at the center is smaller, making it difficult for the resin matrix to penetrate the entire pore structure when constructing the DRCs, therefore existing the gaps in DRCs and weaken the properties. Additionally, porous silica has low loading fraction in DRCs due to its high surface area, which can give rise to a series of problems such as limited mechanical properties [36]. Therefore, it may be suitable to use as the coating layer on UHA particles to construct a structure–function-integrated filler and to use as a reinforced filler for DRCs.

Herein, we synthesized UHA as the starting material [26], followed by performing surface modification on these UHA materials by coating a porous silica (PS) layer via a simple template method, where the thickness of the PS coating layer can be precisely controlled by adjusting the reaction conditions. In comparison with the previous surface modification, the presence of PS layer anchored onto the UHA surface enables the particles to exhibit a dual physical micromechanical interlocking structure within DRCs. This structure consists of the overall 3D urchin-like architecture of the UHA particles, which penetrates into the resin matrix, and the outer porous coating on each whisker of the UHA particles, which allows the resin matrix to infiltrate the pore channels. Together, these features effectively enhance the filler–resin interfacial interaction. Additionally, the coating process occupied the hydroxyl groups on the surface of UHA particles and the PS coating layer reduces the water absorption and dissolution characteristics of UHA, all of which can improve the corresponding properties of corresponding DRCs, especially for the mechanical properties and water sorption/solubility properties. Furthermore, based on the results of cell activity, differentiation and mineralization, these DRCs showed excellent biological activity. This study highlights the benefits of physical interlocking structure and surface-modified UHA, which could provide insights for producing composites with superior performance.

## 2. Materials and Methods

### 2.1. Materials

Ethylenediaminetetraacetic acid disodium salt (EDTA.2Na), calcium nitrate tetrahydrate (Ca(NO_3_)_2_·4H_2_O), disodium hydrogen phosphate dodecahydrate (Na_2_HPO_4_·12H_2_O), sodium hydroxide (NaOH), ammonium hydroxide (28–30%), tetraethyl orthosilicate (TEOS), urea, ethanol, and cyclohexane were all bought from Sinopharm Chemical Reagent Co., Ltd. (Shanghai, China). Cetyltrimethylammonium bromide (CTAB), bisphenol A glycerolate dimethacrylate (Bis-GMA), triethylene glycol dimethacrylate (TEGDMA), camphorquinone (CQ), and ethyl 4-dimethylamino benzoate (4-EDMAB) were purchased from Sigma-Aldrich. Sodium chloride (NaCl), sodium bicarbonate (NaHCO_3_), potassium chloride (KCl), dipotassium hydrogen phosphate trihydrate (K_2_HPO_4_·3H_2_O), magnesium chloride (MgCl_2_), calcium chloride (CaCl_2_), and sodium sulfate (Na_2_SO_4_) were purchased from Shanghai Aladdin Bio-Chem Technology Co., Ltd. (Shanghai, China). The human dental pulp cells (hDPCs) were obtained from dental pulp tissue provided by Shanghai Ninth People’s Hospital (Shanghai, China), based on the informed consent of patients. Dulbecco’s modified eagle’s medium (DMEM), fetal bovine serum (FBS) and trypsin were purchased from Gibco. Penicillin-streptomycinamphotericin B solution, phosphatic buffer solution (PBS), Calcin-AM/PI Double Staining Kit and Cell Counting Kit-8 (CCK-8) were purchased from Beyotime. All chemicals were used without further purification.

### 2.2. Synthesis of UHA/PSx Particles

**Figure 1:**
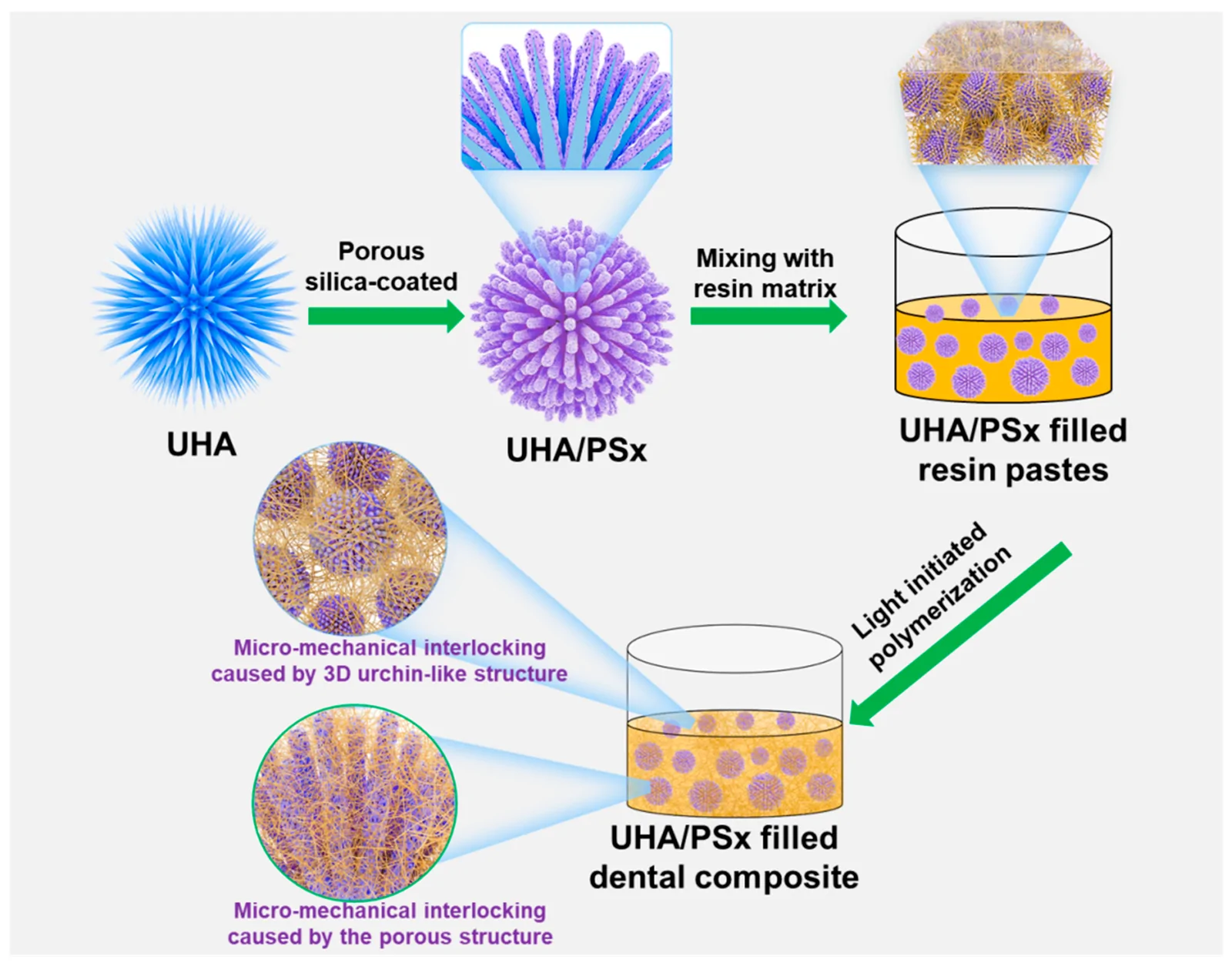
Schematic illustration of the synthesis of UHA/PSx particles used in dental composites.

UHA particles were synthesized according to a facile hydrothermal precipitation technique following a previous study [5]. The synthesis process of UHA/PSx particles is illustrated in Figure 1, where the porous silica shell was evenly coated onto each whisker surface of UHA. Briefly, UHA particles (0.2 g) were dispersed into deionized water (30 mL) to form solution 1. Meanwhile, CTAB (0.18 g) and urea (0.18 g) were added to deionized water (30 mL) to obtain solution 2. Subsequently, solution 1 was introduced into solution 2 and stirred at 60 °C for 1 h. Then, a solution consisting of TEOS (1.11 mL) and cyclohexane (20 mL) was slowly added into the above mixture and the reaction was performed at 60 °C with stirring for a fixed time period (3, 5, 7, and 10 h, respectively). The white products were collected through centrifugation and washed with water and then with absolute ethanol for three times. Finally, the obtained products were dispersed into acetone and refluxed at 60 °C for 24 h, a process which was repeated three times, to remove the CTAB templates [7]. The white powder was obtained after washing with absolute ethanol and drying in vacuum at 80 °C. Based on the different reactive times, the finally powder materials were named as UHA/PS3, UHA/PS5, UHA/PS7, and UHA/PS10, respectively, and were to be used as fillers in dental resin composites.

### 2.3. Preparation of Dental Resin Composites (DRCs)

Firstly, the resin matrix was made by mixing monomer (49.5 wt% Bis-GMA and 49.5 wt% TEGDMA) with photo-initiation system (0.2 wt% CQ and 0.8 wt% 4-EDMAB) in the dark. Afterward, each filler was completely mixed with the resin matrix through a three-roll mill (EXAKT 80 E Plus, Norderstedt, Germany) to obtain the composite pastes, and the pastes were further inserted into corresponding silicon rubber and cured with a curing lamp for 60 s on each side.

### 2.4. Characterization

#### 2.4.1. Morphology and Crystal Structure of UHA/PSx

The morphology-structure of UHA/PSx particles was evaluated by field emission scanning electron microscope (FE-SEM, SU8010, Tokyo, Japan) and transmission electron microscopy (TEM, JEOL JEM-2100 F) operated at 5 kV and 200 kV, respectively. Elemental mapping was further performed by using an analytical TEM equipped with an energy dispersive spectroscopy (EDS) system. The crystalline structure was analyzed using XRD (D/max-2550VB+/PC, Tokyo, Japan). The porous structure was evaluated by small-angle X-ray scattering (SAXSess mc2, Graz, Austria). The porosity and specific surface area were evaluated with N_2_ adsorption/desorption isotherms (Quadrasorb-SI, Boynton Beach, FL, USA) at 77 K, and the samples were degassed under vacuum at 100 °C for 12 h before measurements. The compositional elements of samples were measured by X-ray photoelectron spectroscopy (XPS, Escalab 250Xi, Waltham, MA, USA). The amount of silica on the surface of TUSHA-n was further quantitatively determined based on JY/T 015–1996 inductively coupled plasma atomic emission spectrometry (ICP-AES, Prodigy-ICP, Hudson, NH, USA).

#### 2.4.2. Mechanical Properties of UHA/PSx-Filled DRCs

Based on ISO-4049 [38], the mechanical properties of various DRCs were evaluated using a universal testing machine (Instron 5969), including flexural strength, flexural modulus, and compressive strength. Specifically, each uncured composite paste was filled into the molds with the sizes of 25 mm × 2 mm × 2 mm (flexural test) and Φ 4 mm × 6 mm (compressive test) and then cured for 60 s on both sides (n = 6). The crosshead speed of the machine was 0.75 mm/min.

#### 2.4.3. Fracture Surface of UHA/PSx-Filled DRCs

The samples were collected after the flexural test and used for observing the fractured morphology surface of each DRC by SEM.

#### 2.4.4. Water Sorption and Solubility of UHA/PSx-Filled DRCs

The water sorption and solubility of DRCs were evaluated according to ISO 4049-2019 [38] and previous studies [34]. All the cured specimens (Φ 15 mm × 1 mm, n = 4) were placed in an oven at 37 °C for 22 h and then transferred to the desiccator for 2 h. This process was repeated until a constant mass (m1) was obtained. Subsequently, the specimens were soaked in deionized water at 37 °C for 7 days and then dried with filter paper, and weighed (m2). This drying process was repeated until the constant mass (m3) was obtained. Finally, the volume of each specimen was calculated based on their mean diameter and thickness. The water sorption (W_sp_) and solubility (W_sl_) of each specimen was calculated with the formulas of W_sp_ = (m2 − m3)/V and W_sl_ = (m1 − m3)/V, where the units of m and V are μg and mm^3^, respectively.

#### 2.4.5. Curing Depth of UHA/PSx-Filled DRCs

Based on ISO 4049-2019 [38], each composite paste was placed in a cylindrical mold (Φ 4 mm × 10 mm, n = 3) and irradiated vertically against the paste surface on one side using a light curing unit for 20 s. The sample was demolded and the softer uncured material was removed. The light-curing depth value was determined using a vernier caliper by dividing the measurement by 2.

#### 2.4.6. Mineralization of DRCs Surfaces

Various cured specimens (Φ10 mm × 1 mm) were immersed in 15 mL of SBF (pH = 7.4) at 37 °C for 1, 7, and 15 days to assess their surface mineral deposition. At each time point, the specimens were carefully extracted, gently rinsed with deionized water, and subsequently dried in a drying oven set at 40 °C for 24 h. The mineralized layer on the surfaces of the samples was examined using FE-SEM.

#### 2.4.7. In Vitro Cell Activity of UHA/PSx-Filled DRCs

1. Human dental pulp stem cells (hDPSCs) culture

Healthy teeth were collected from Shanghai Ninth People’s Hospital affiliated with Shanghai Jiaotong University School of Medicine after informed consent. Firstly, the teeth were dissected to expose the pulp tissues in a sterile environment. The pulp tissue was then separated, cut into small pieces and pressed with a coverslip in a Petri dish. Subsequently, cell culture medium containing Dulbecco’s Modified Eagle’s Media (DMEM), 10% fetal bovine serum (FBS), and 1% penicillin/streptomycin was added into the above dish and placed at 37 °C under a 5% CO_2_ atmosphere. Finally, the hDPSCs crawled out of the tissue block, and the cells of generation 3–6 were used in this study.

2. Preparation of DRCs extracts

DRC disk pieces (Φ 10 mm × 1 mm, n = 6) were prepared, sterilized by UV irradiation on each side, and washed with PBS. Subsequently, these disks were immersed in the medium (containing 89% DMEM, 10% FBS, and 1% penicillin/streptomycin) for 24 h at 37 °C to obtain the specimen extracts. The ratio of the volume of culture medium to the surface area of the specimen was 3 cm^2^/mL, according to the ISO-10993 standard [39].

3. Cytotoxicity and cell proliferation

In vitro cytotoxicity of DRCs was determined by the CCK8 assays. Briefly, the hDPSCs were seeded in 96-well plates (10^3^ cells/well) and cultured with DRCs extract (100 mL/well) for 1, 3, 5, and 7 days. After each time point, CCK-8 assays were performed and cell cytotoxicity was determined by detecting the optical density of solution at 450 nm using an enzyme labeling instrument (TECAN Spark, Männedorf, Switzerland). In addition, the hDPSCs were seeded in 48-well plates (10^4^ cells/well) and the Calcein-AM/PI Double Stain Kit (Yeasen, Shanghai, China) was used to conduct the Live/Dead assay, and the cells morphology was observed using a fluorescence microscope (Olympus, Tokyo, Japan). The surface coverage of the different fluorescence was analyzed by ImageJ software Ver: 1.54q14.

4. Alkaline phosphatase (ALP) activity test

The hDPSCs were cultured with extracts of DRCs for 14 days, and a blank control was set. For ALP staining, the cells were fixed with 4% paraformaldehyde for 30 min. Subsequently, the fixative was removed and the cells were stained using BCIP/NBT Alkaline Phosphatase Chromogenic Kit (Beyotime, Shanghai, China). The staining images were observed by an inverted microscope (Nikon, Japan). For the ALP activity assay, after lysis with 1% Triton X-100 (Beyotime, China) for 30 min, the ALP protein of the hDPSCs was measured by an Alkaline Phosphatase Assay Kit (Beyotime, China) and recorded as the OD value at 405 nm. Next, the total protein concentration in each sample was determined using a BCA protein assay kit (Thermo, Waltham, MA, USA) for normalization.

5. Real-time polymerase chain reaction (RT-PCR) analysis

The expression of mineralization-related genes in hDPSCs was detected by RT-PCR. After incubating the hDPSCs with DRCs extracts for 14 days, RNA was isolated using TRIzol (Invitrogen, Carlsbad, CA, USA). After measuring the RNA concentration and purity with a NanoDrop™ 1000 ultraviolet–visible spectrophotometer (Thermo Scientific, Waltham, MA, USA), the RNA was converted to cDNA using a PrimeScriptTM RT kit (Takara, Shiga, Japan). Subsequently, RT-PCR for ALP and osteopontin (OPN) was performed using a LightCyler 480 (Roche, Switzerland) and SYBR Green (Yeasen, Shanghai, China). Expression data were normalized to GAPDH as the internal control, and relative quantification was performed using the 2−ΔΔCt method with three technical replicates and three independent biological replicates to ensure reproducibility. Assays for dentin matrix protein-1 (DMP-1) and dentin sialophosphoprotein (DSPP) genes were performed using hDPSCs conditioned and cultured for 14 days in the same manner as described above. All primer sequences are provided in Supplementary Table S1.

### 2.5. Statistical Analysis

All data in the figures were demonstrated as means with standard deviation (mean ± SD). The statistics were analyzed using Origin software or GraphPad Prism software Ver.: 10.5.0. The statistically significant values were evaluated using two-sided Student’s *t*-tests and one-way analysis of variance (ANOVA) tests, with the statistical significance set at * *p* < 0.05, ** *p* < 0.01.

## 3. Results and Discussion

### 3.1. Morphology of UHA/PSx

**Figure 2:**
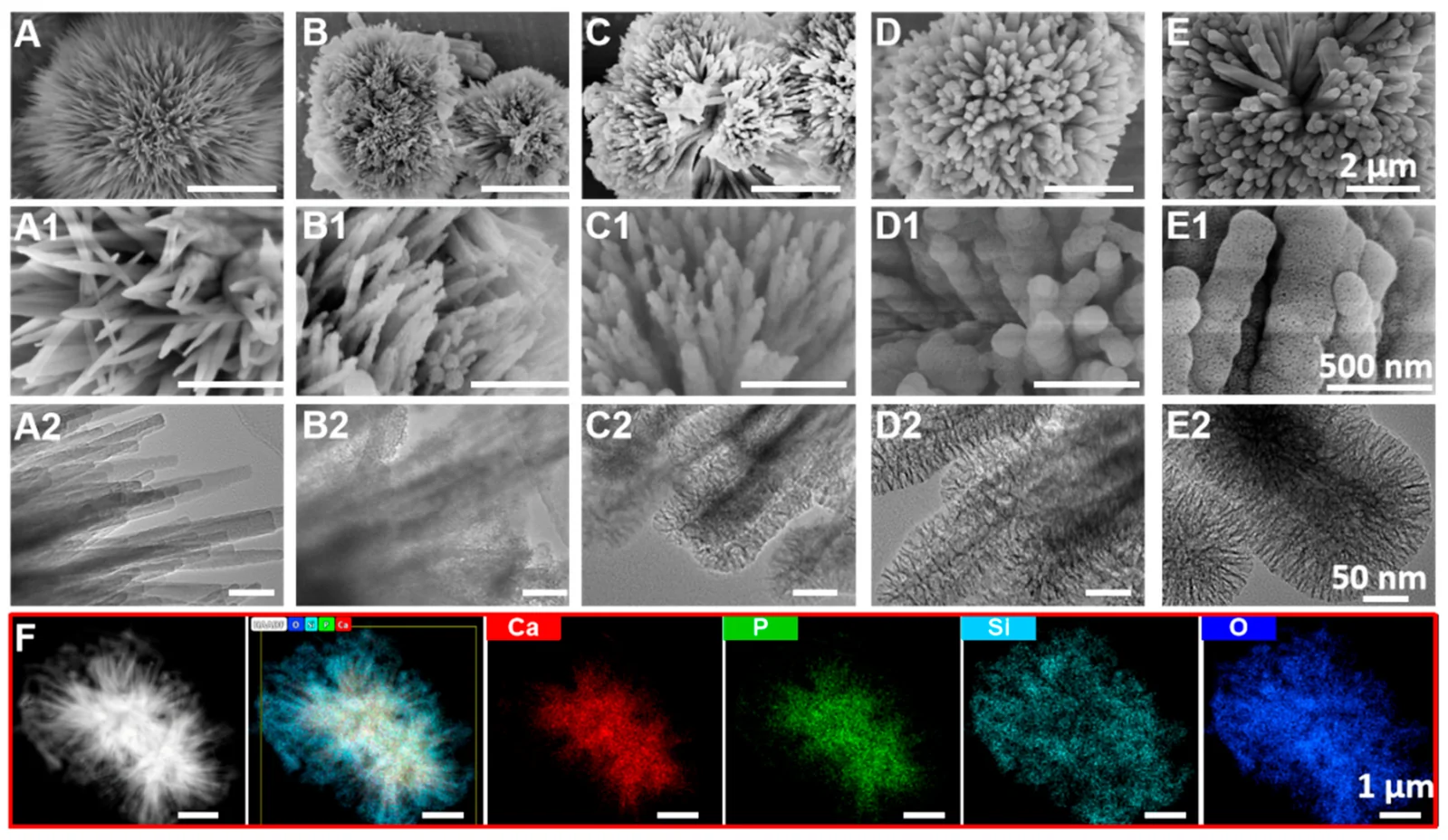
SEM (**A1**–**E1**) and TEM (**A2**–**E2**) images of the UHA/PSx particles with various porous thicknesses prepared at different times: 0 h (**A**,**A1**,**A2**, denoted as UHA); 3 h (**B**,**B1**,**B2**, denoted as UHA/PS3); 5 h (**C**,**C1**,**C2**, denoted as UHA/PS5); 7 h (**D**,**D1**,**D2**, denoted as UHA/PS7), and 10 h (**E**,**E1**,**E2**, denoted as UHA/PS10); TEM image and corresponding elemental mapping (**F**) of the representative UHA/PS5.

The morphologies of UHA/PSx particles were investigated by SEM and TEM images, and the results are presented in Figure 2. The particles before and after surface coating showed urchin-like hierarchical appearance comprising one dimensional continuous parallel array nanorods, indicating that the surface coating process did not change the morphology of particles (Figure 2A). In comparison, each nanorod in UHA particles presented a smooth surface, while the UHA/PSx particles exhibited obvious rougher surfaces caused by porous silica (PS) coating (Figure 2B–E). In addition, as shown in the SEM images with high magnification (Figure 2(B1–E1)) and TEM images (Figure 2(B2–E2)), the PS was evenly coated on the surface of each nanorod, and the coating thickness of PS was controlled by the reaction time, which was increased with a longer reaction time. For the analysis of their elemental composition, UHA/PS3 was selected as the representative particles. Its chemical compositions and element distributions was evaluated by scanning transmission electron microscope (STEM) image and the corresponding elemental mapping (Figure 2F). The results demonstrate that the *Ca*, *P*, *O*, and *Si* elements simultaneously homogeneously distributed in the particle. More importantly, compared with *Ca* and *P* elements, the *Si* and *O* elements were homogeneously distributed at the edge of this particles, also demonstrating the PS was evenly coated on the surface of the particle.

### 3.2. Structure of UHA/PSx

**Figure 3:**
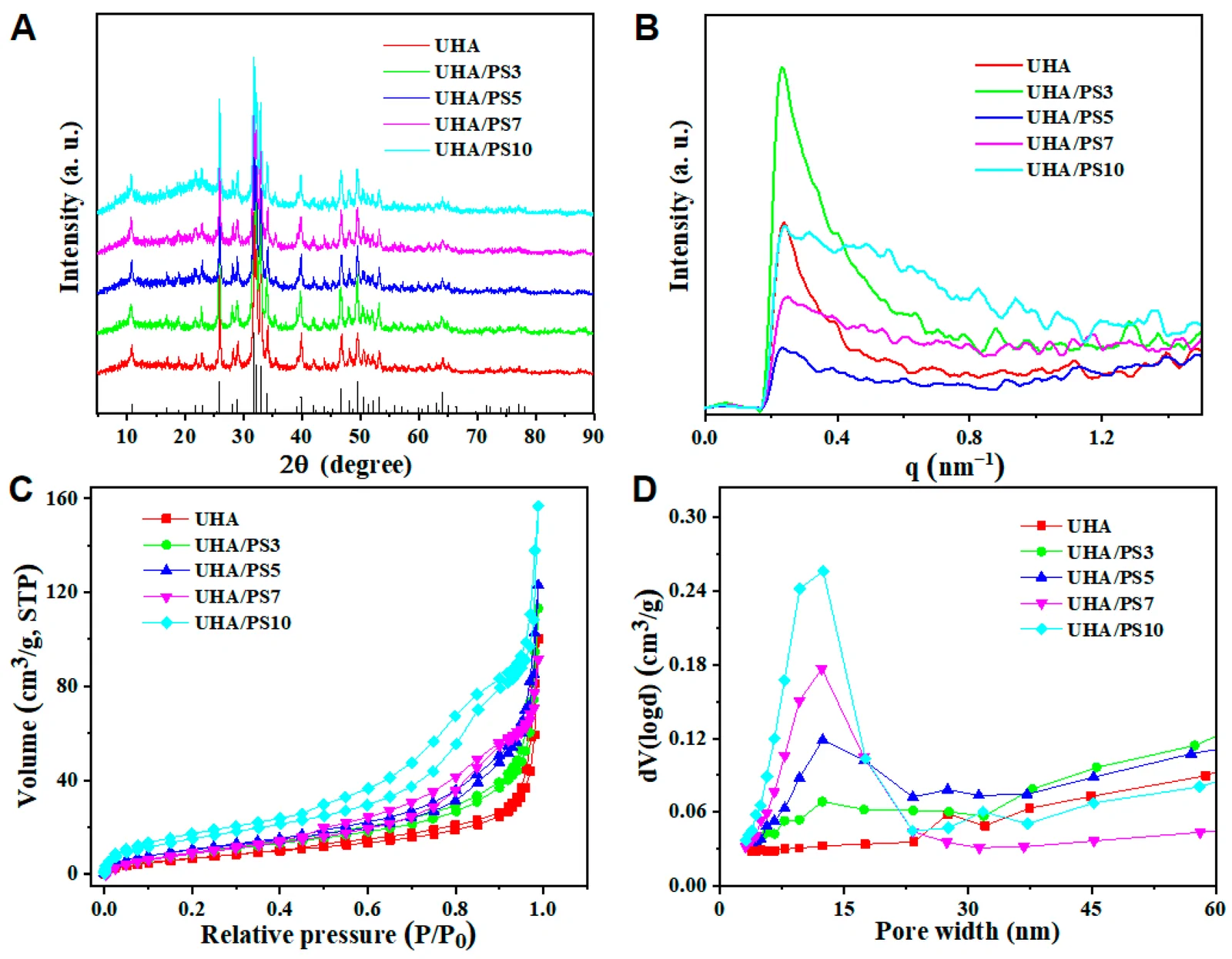
X-ray diffraction patterns (**A**), small angle X-ray scattering patterns (**B**), N_2_ adsorption–desorption isotherms (**C**), and pore size distributions (**D**) of UHA/PSx particles.

As shown in XRD patterns in Figure 3A, the characteristic peak of UHA and UHA/PSx agrees with the standard card of HAp phase (ICDD 09–0432) [25], which indicated that the PS coating did not influence the crystalline structure of HAp. Compared with UHA, a broad diffraction peak at about 23° was found in UHA/PSx, which corresponded to the amorphous structure of silica. This result demonstrated that the PS was successfully coated onto the UHA particle. In addition, the relative intensity of this broad diffraction peak became stronger with a larger “x” value, indicating the coating amount of PS was gradually increased with the reaction time, which agreed well with the above SEM and TEM images.

The porous structure of UHA/PSx particles was firstly assessed with SAXS. As shown in Figure 3B, more than one scattering peak was observed in the SAXS patterns of all particles, which indicated that there is a hierarchical porous structure in these particles [40,41]. In addition, for the UHA/PSx particles, the hierarchical porous structure was gradually apparent with the increase in the “x” value, mainly due to the presence of the PS coating. We further evaluated the porous structure of these particles by N_2_ adsorption/desorption measurements. As shown in Figure 3C, the isotherms of all particles do not level off in the vicinity of P/P_0_ = 1, indicating the presence of the macropores [42]. Meanwhile, all the particles exhibit a similar type IV isotherm and a typical H3-hysteresis loop in 0.4 < P/P_0_ < 1.0 suggesting the existence of various sized, slit-shaped mesopores in the materials [43], which mainly attributed the PS coating on the UHA surface. In comparison, the BET surface area and cumulative pore volume values of these particles are proportional to the PS coating time on the UHA surface, and the UHA/PS10 particle presented the largest specific surface area and cumulative pore volume (Table 1). Meanwhile, the DFT pore volume curves with multi-peaks (Figure 3D) further demonstrate the multimodal pore size distributions of these particles [44].

### 3.3. Compositional Elements of UHA/PSx

**Figure 4:**
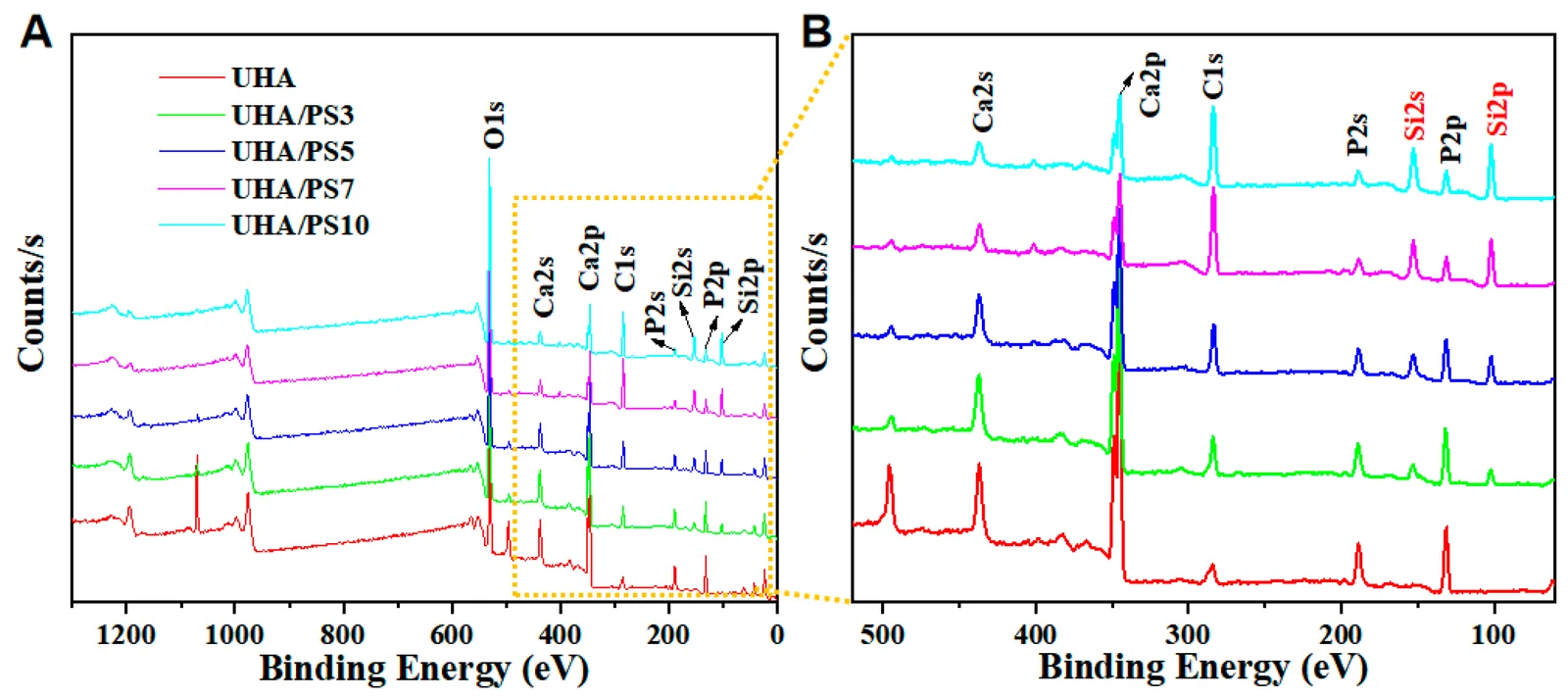
XPS spectra of UHA, UHA/PS3, UHA/PS5, UHA/PS7, and UHA/PS10. (**A**) Survey Spectrum; (**B**) Ca 2s, Ca 2p, C 1s, P 2s, Si 2s, P 2p, Si 2p Spectrum.

The compositional elements of UHA/PSx were investigated by the full XPS spectra. As shown in Figure 4, compared with UHA, all UHA/PSx particles showed the obvious Si element signal, and this signal peak was gradually increased with the “n” value, the atomic percentage was listed in Table 2. These results revealed that the thickness of the PS layer onto UHA surface is controllable, which corresponds to the previous SEM and XRD results (Figure 2 and Figure 3). Furthermore, the content of silica was quantitatively analysis by ICP-AES, where the values were 2.40, 4.67, 13.55, and 28.93 mg/g of UHA/PS3, UHA/PS5, UHA/PS7, and UHA/PS10, respectively, which was consistent with the XPS results (Table 2).

### 3.4. Mechanical Performance of UHA/PSx-Filled RDCs

During the formulation of DRCs, the UHA/PS10 with the thickest PS coating layer presented the lowest maximum filler loading, because of its high surface area. In order to determine the reinforcing effect of various UHA/PSx with different thicknesses of the PS coating layer, all produced DRCs were loaded at 63 wt.%.

**Figure 5:**
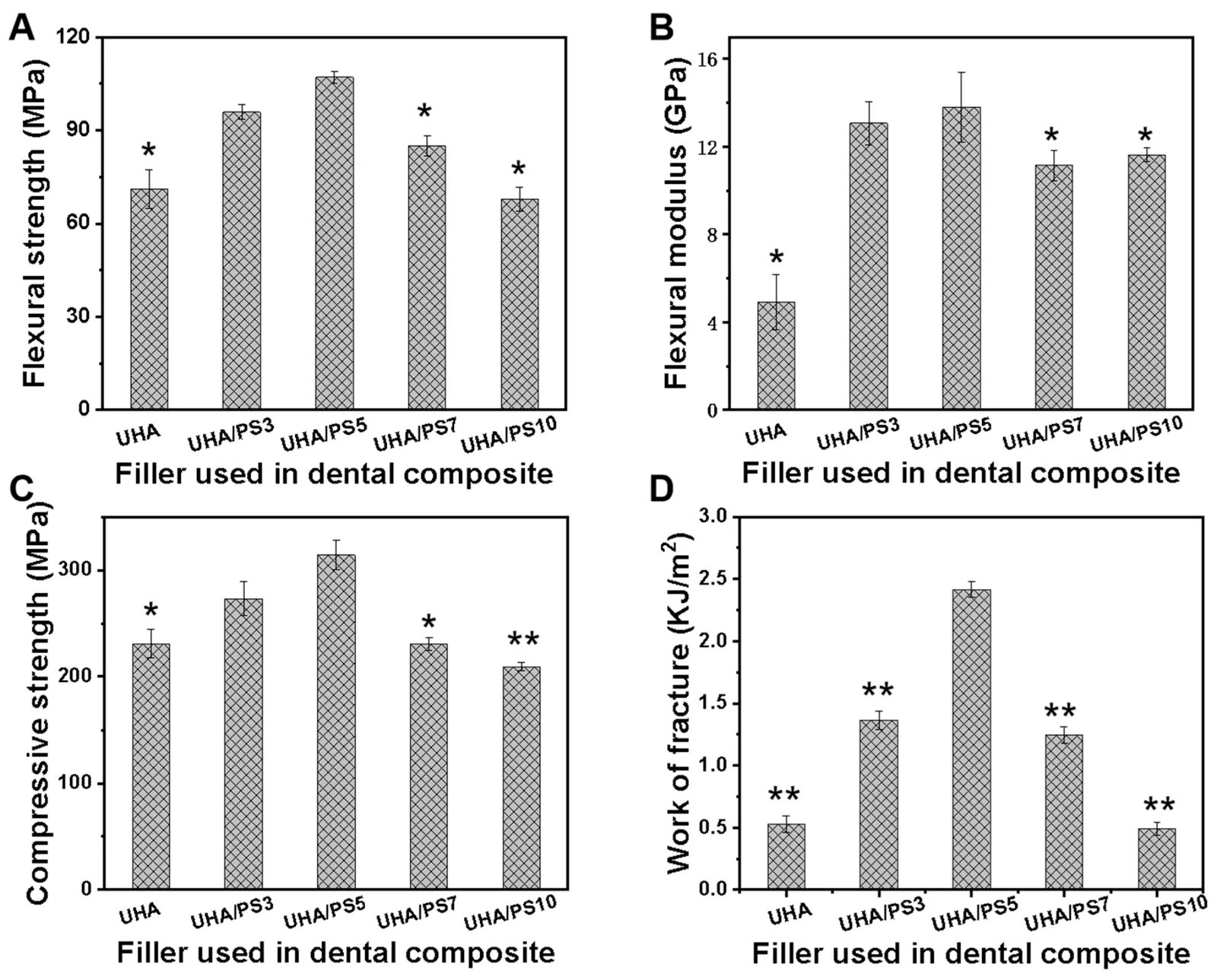
Flexural strength (**A**), flexural modulus (**B**), compressive strength (**C**), and work of fracture (**D**) of dental composites filled with different UHA/PSx particles at 63 wt.% filler loading. * *p* < 0.05 and ** *p* < 0.01, compared with the UHA/PS5-filled composite.

The mechanical properties of DRCs, including flexural strength (FS), flexural modulus (FM), and compressive strength (CS), were measured to evaluate the reinforcing effect of UHA/PSx particles (Figure 5). As expected, compared with the UHA-filled dental composites, UHA/PSx did improve the mechanical performance of corresponding DRCs, where the FS, FM, and CS of UHA/PS5-filled composites increased by 50.7%, 181.6%, and 35.9% compared to UHA-filled composites. In addition, by comparison, the reinforcement effect was affected by the PS coating thickness, where a first increasing and then decreasing trend in both flexural strength and compressive strength is found with increasing PS coating thickness (Figure 5A,C). More specifically, UHA/PS5 exhibited the best reinforcing effect for dental composites, giving large improvements of 12.6%, 5.4%, 15.1%, and 76% for SF, FM, CS, and work of fracture values, respectively, over the UHA/PS3-filled composite, and 59.7%, 19.0%, 50.2%, and 380% for SF, FM, CS, and work of fracture values, respectively, over the UHA/PS10-filled composite. These results could be attributed to that filler–resin matrix interface interaction in UHA-filled was achieved by the single micromechanical interlocking structure between the whisker of UHA and resin matrix, while the whisker and porous structure in PS coating layer of UHA/PS5 fillers can form the dual micromechanical interlocking structure with resin matrix. However, for UHA/PS10, the overlapped PS resulted in the undesirable matrix/filler adhesion (Figure 2G,H), where the resin matrix cannot be embedded in the entire porous of PS layer, and thereby decreased the mechanical performance of composites.

### 3.5. Fractured Morphology of UHA/PSx-Filled DRCs

**Figure 6:**
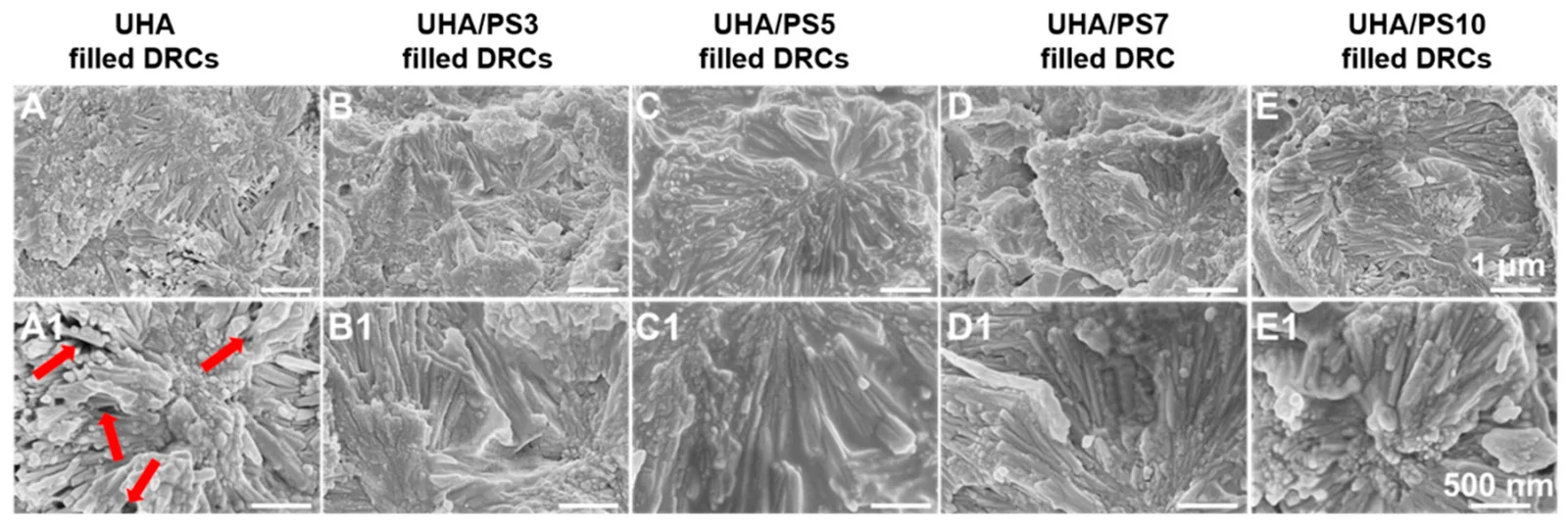
Fractured morphology of UHA-filled dental resin composite (**A**,**A1**), UHA/PS3-filled dental resin composite (**B**,**B1**), UHA/PS5-filled dental resin composite (**C**,**C1**), UHA/PS7-filled dental resin composite (**D**,**D1**), and UHA/PS10-filled dental resin composite (**E**,**E1**).

The fractured morphologies of UHA/PSx-filled DRCs were examined to assess the fillers/resin matrix interfacial bonding and the effect of PS coating contributing to the fracture mechanism of the composites. As shown in Figure 6, for the UHA-filled composite, there are obvious gaps at the fillers/resin matrix interface (Figure 6A,A1), which was associated with the weak filler–resin matrix adhesion as a result of the absence of the PS coating onto the UHA surface. In comparison, the UHA/PSx fillers were tightly embedded in the resin matrix and had almost no gaps between fillers and resin, which mainly attributed to the dual micromechanical interlocking formed by the whisker structure of the UHA particles and porous structure of the PS coating layer. Specifically, UHA/PS5 particles were tightly embedded into the resin matrix just like roots growing in the soil (Figure 6C,C1), demonstrating that more energy would be consumed during the strength tests of this composite, which is an essential in enhancing the mechanical performance [45,46]. These results further confirm that the PS coating layer with a suitable thickness on the UHA surface effectively improved the matrix/filler interfacial bonding and the mechanical performance of dental composites.

### 3.6. Water Sorption and Solubility of UHA/PSx-Filled DRCs

**Figure 7:**
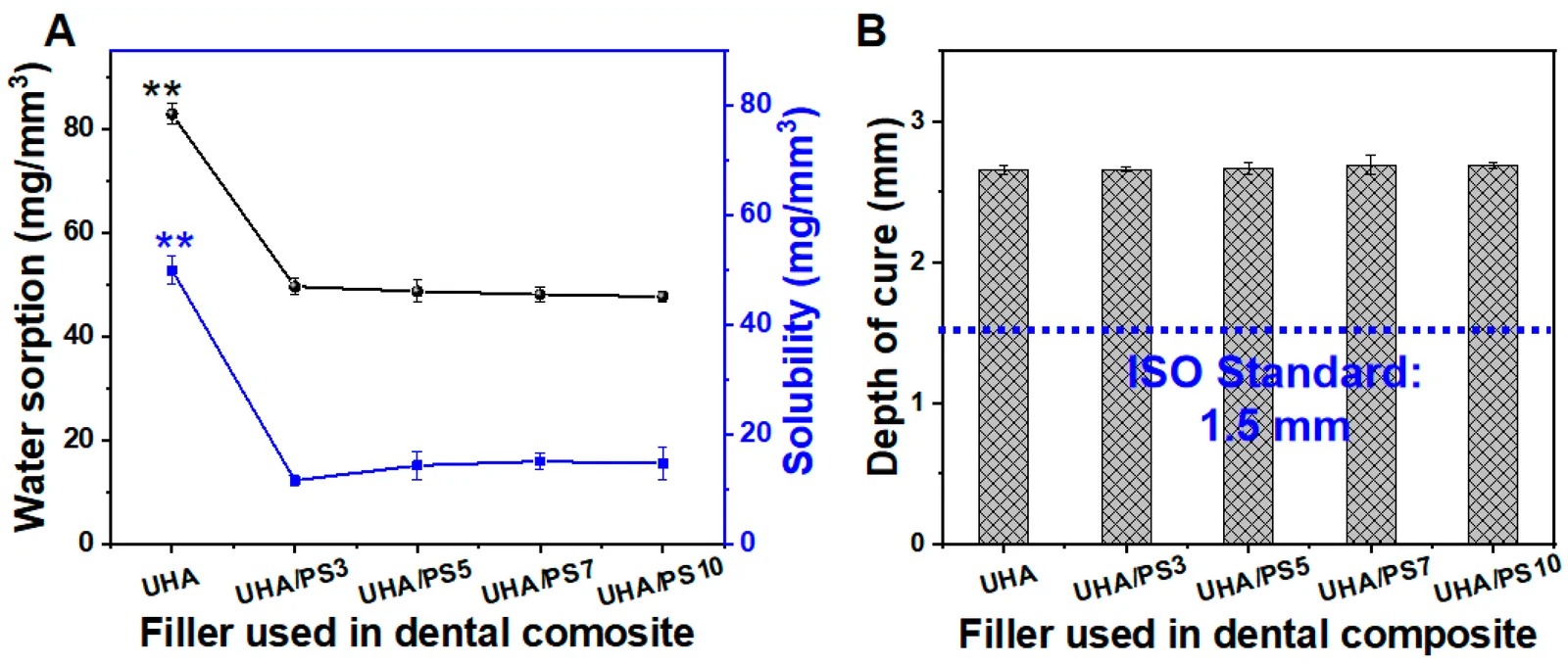
Water absorption and solubility properties (**A**) and curing depth (**B**) of UHA/PSx-filled dental resin composites. ** *p* < 0.01, compared with the UHA/PS5-filled composite.

The oral environment is moist, which requires dental composites to have excellent stability in wet, body-temperature environments. The low absorption and solubility of dental composites are essential for the clinical applications, where the absorbed water would weaken the filler–resin matrix interfacial interaction and the solubility would result in their demineralization, which finally lead to the failure of restoration [47,48]. Therefore, based on the ISO 4049-2019, the water absorption and solubility properties of UHA-filled dental composites and various UHA/PSx-filled dental composites were measured after soaking in water at 37 °C for 7 days. As shown in Figure 7, compared with UHA-filled dental composites, the water absorption and solubility values of UHA/PSx-filled dental composites presented a significant decrease, which mainly attributed to the PS coating layer on UHA surface, enhancing the stability and filler/resin matrix interface bonding of UHA/PSx versus UHA. The UHA particles contains a large number of hydroxyl groups, resulting in its high water absorption. By contrast, the PS coating layers in UHA/PSx particles not only take up the hydroxyl group of UHA particle during the modification process, but also acts as a hydrophobic coating to prevent UHA from absorbing water. Specifically, the water sorption and solubility values of various UHA/PSx-filled dental composites showed no significant difference, and the values of UHA/PS5-reinforced composites are decreased by 41.3% and 71.6% compared with those of UHA, respectively.

### 3.7. Curing Depth of UHA/PSx-Filled DRCs

A higher curing depth of DRCs is also crucial for their clinical application, offering convenience for practitioners and reducing the treatment time [49]. As shown in Figure 7B, the curing depth value of each experimental DRC was far exceeded the requirement of ISO 4049-2019 (1.5 mm). Additionally, the PS coating layer and its coating thickness has no effect on the curing depth values, which mainly attributed to the lower refractive index (~1.46) of SiO_2_ than hydroxyapatite (~1.62) [10].

### 3.8. Remineralization of UHA/PSx-Filled DRCs

**Figure 8:**
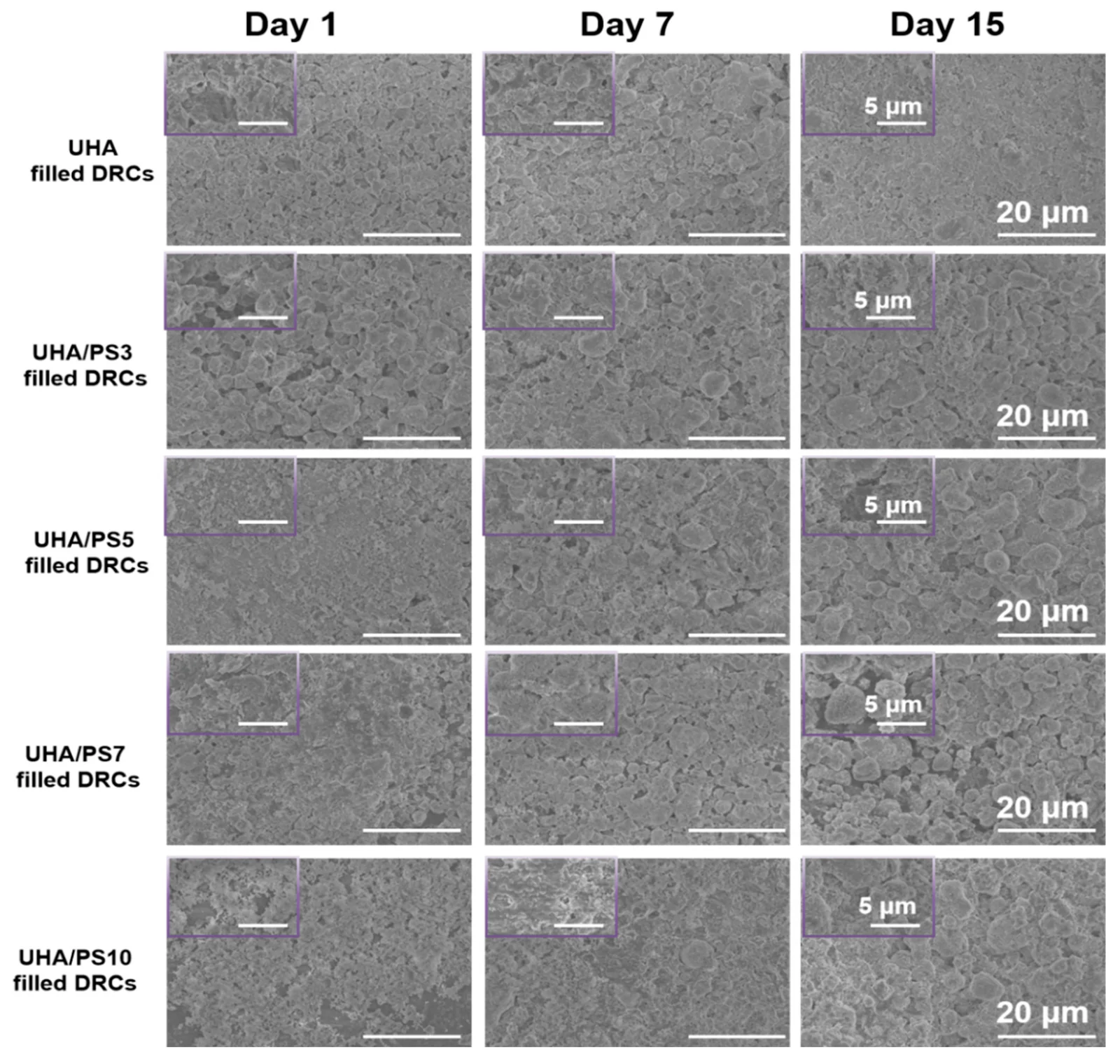
FE-SEM images of the surface morphology of UHA/PSx-filled dental resin composite after soaking in SBF for 1, 7, and 15 days.

The in vitro remineralization activity of the UHA/PSx-filled DRCs can promote the formation of an apatite layer on their surface when immersed in SBF, which is effective to reduce the DRC tooth space caused by photo-polymerization, and thus reduce bacteria aggregation [50]. Herein, the surface morphology and Ca and P elements concentration of the remineralization layer of UHA/PSx-filled DRCs was evaluated after immersion in SBF for 1, 7, and 15 d (Figure 8), and the UHA-filled DRCs was used as the control group to investigate the effect of the existence of the PS coating layer on the remineralization activity. After 1 day of immersion, the obvious mineralization layers of various DRCs were clearly observed, and the mineralization layer becomes denser as the immersion time increases to 7 and 15 days. Additionally, it is interesting to observe that no obvious difference was found between UHA and various UHA/PSx-filled DRCs, indicating that the existence of PS layer did not affect the in vitro remineralization activity. Based on the previous studies, the fillers used in DRCs containing calcium and phosphorus can promote the formation of apatite remineralization layer, which has the potential to promote the deposition of Ca^2+^ and PO^3−^ on the diseased enamel and therefore maintain the dynamic balance of minerals in enamel [51]. Hence, in this study, the PS coating layer onto the whisker of UHA filler has no effect on the mineralization-promoting properties of calcium and phosphorus.

### 3.9. Cell Viability of UHA/PSx-Filled DRCs

**Figure 9:**
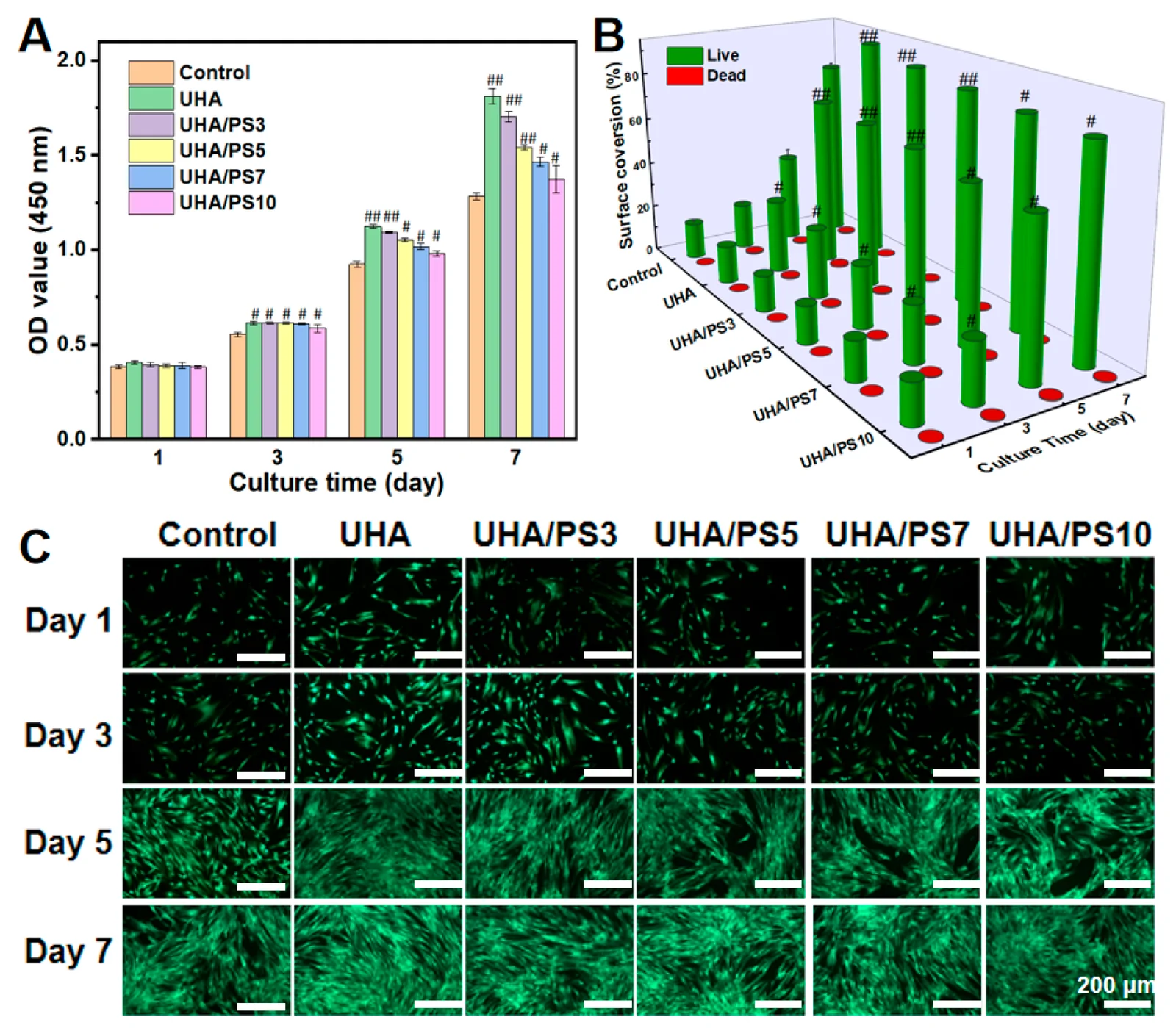
HDPC viability cultured onto DRCs filled with UHA, UHA/PS3, UHA/PS5,UHA/PS7, and UHA/PS10 for 1, 3, 5, and 7 days (**A**), fluorescence microscopy images of HDPCs cultured onto these composites (**B**), live/dead cell percentage area of these materials based on fluorescent images using Image J software (**C**). The green color indicates living cells, while the red color indicates dead cells. # *p* < 0.05, ## *p* < 0.01, compared with the control (Blank) group.

The biocompatibility of various DRCs is vital due to their application in the complex oral environment [52,53]. The cell viability of DRCs filled with different UHA/PSx was evaluated with CCK-8 assay. As shown in Figure 9A, the OD values of all DRCs show a gradual increase from day 1 to day 7, indicating their well biocompatibility. Additionally, the OD values at 3, 5 and 7 days of all UHA/PSx-filled DRCs are significantly higher than the blank group, especially for the UHA/PS3 and UHA/PS5-filled DRCs. This result indicated that UHA/PSx-filled DRCs can enhance cell proliferation, which maybe mainly attributed to the fact that UHA and PS coating layer were biologically active components. Extracts from these materials contain calcium and fluorine ions, which have been reported to favor the proliferation of hDPSCs [54]. Furthermore, when the culture time increase to 7 days, the OD value of UHA/PS7 and UHA/PS10-filled DRCs was lower than UHA/PS3 and UHA/PS5, indicating their relatively weak cell viability. This result may mainly be due to the weaker biological activity of PS coating layer than UHA whisker.

The morphology of cell cultured onto these DRCs as a function of culture time is also evaluated and the fluorescent images results were shown in Figure 9C. Live spindle-shaped HDPCs are clearly observed in various DRCs during different culture periods by the LIVE/DEAD assay, and the number of gradually increases with increasing the culture time. In particular, there are almost no dead cells observed after 7 days of culture, which corroborates the results of CCK-8 assay. Additionally, as shown in Figure 9B, the surface coverage occupied by live/dead cells were calculated with ImageJ software based on the live/dead staining fluorescence images, which shows the same trends with the CCK-8 results. These results indicate that the PS coating layer on the UHA surface does not affect cell growth or the proliferation of corresponding DRCs.

### 3.10. In Vitro Mineralized Differentiation of hDPSCs

**Figure 10:**
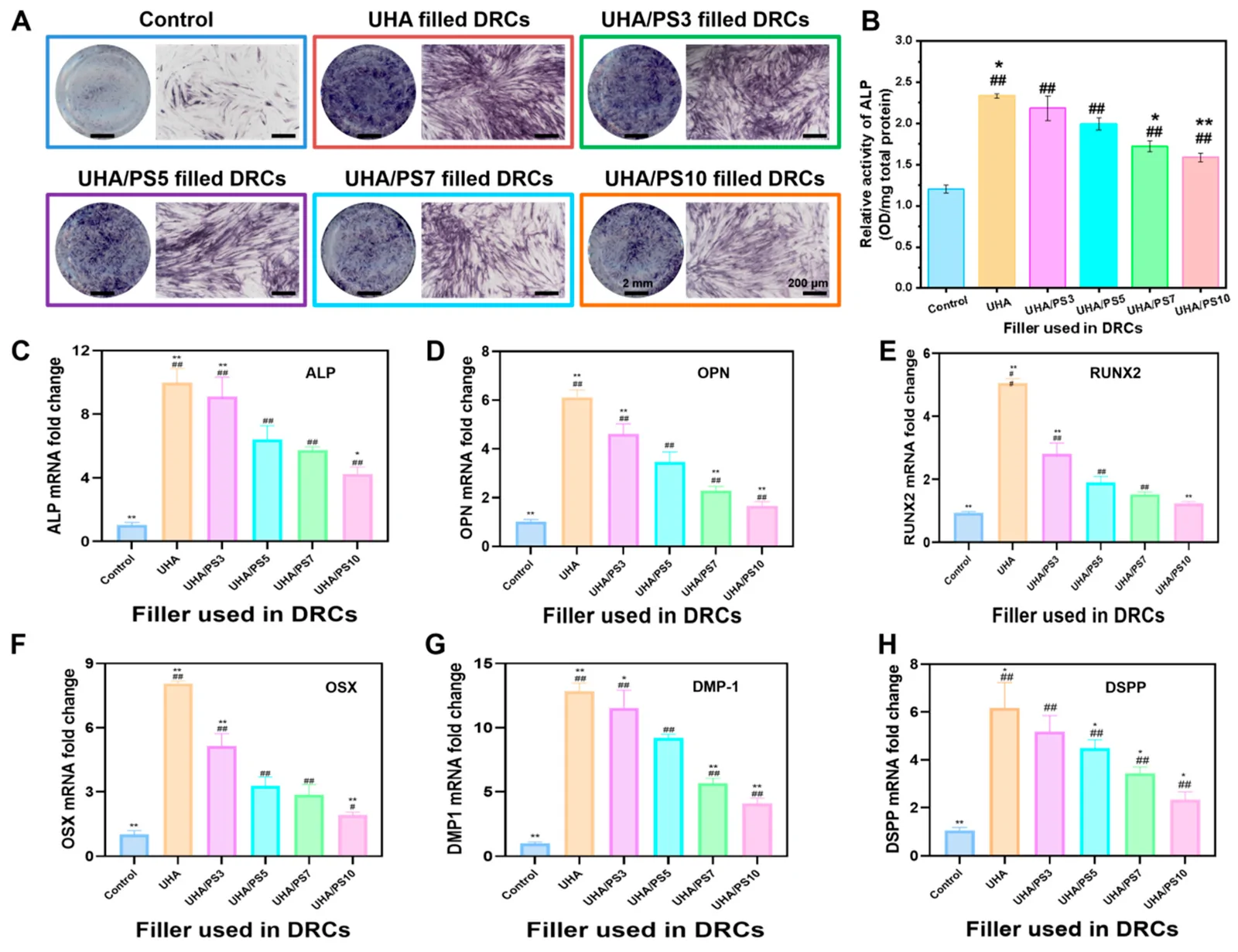
Mineralized differentiation of hDPSCs in vitro. (**A**) ALP staining of hDPSCs and (**B**) semiquantitative analysis of ALP activity in hDPSCs cultured in DRCs filled with UHA, UHA/PS3, UHA/PS5,UHA/PS7,or UHA/PS10 extracts medium for 14 days. (**C**–**H**) The mRNA expression in hDPSCs cultured in DRCs extracts medium for 14 days. # *p* < 0.05, ## *p* < 0.01, compared with the control (Blank) group, * *p* < 0.05, ** *p* < 0.01, compared with the UHA/PS5-filled DRCs group.

To further evaluated the cells proliferation properties of UHA/PSx-filled DRCs, the in vitro mineralized differentiation were also evaluated. As shown in Figure 10, after 14 days, ALP staining resulted varied significantly between the blank group and experimental groups (Figure 10A). Compared with blank group, hDPSCs cultured with UHA/PSx-filled DRCs extracts displayed deeper staining. Quantitative analysis of ALP protein, following cell lysis (Figure 10B), revealed significantly higher ALP expression in the UHA/PSx-filled DRCs groups compared to blank group. Additionally, UHA-filled DRCs presented the best ALP expression among all the experimental groups, indicating that the PS coating layer has weak influence on the expression performance of ALP. As ALP is an early indicator of mineralized differentiation [55], staining and quantitative analysis of ALP showed that DRCs containing UHA could achieve mineralization induction of hDPSCs by body fluid. Furthermore, genes associated with mineralization and odontogenic differentiation were detected by RT-PCR, specifically the ALP, OPN, DMP-1, RUNX-2, OSX, and DSPP genes. Among them, DMP-1 and DSPP play crucial roles in dentin biomineralization, and ALP, OPN, RUNX-2, and OSX are typical biomarkers of osteogenesis and odontogenesis [56]. For the ALP gene (Figure 10C), up-regulation of its mRNA was achieved at 14 days using DRCs extracts of the experimental groups, especially UHA and UHA/PS3-filled DRCs, aligning with the results of ALP staining. Additionally, the experimental groups of DRCs also induced an up-regulation of the OPN gene at 14 days, also especially in the UHA and UHA/PS3-filled DRCs groups (Figure 10D). RUNX2 and OSX expression (Figure 10E,F) were also elevated, indicating enhanced odontogenic transcriptional activity. Moreover, DMP-1 (Figure 10G) and DSPP (Figure 10H) were significantly up-regulated in the UHA and UHA/PS3 groups, further supporting dentin biomineralization and pulp protection. Taken together, the coordinated up-regulation of these markers reflects enhanced odontogenic differentiation, dentin matrix formation, and pulp–dentin complex protection, thereby confirming the potential of UHA/PSx-filled DRCs for dentin regeneration.

In addition, the mineralized differentiation of cells gradually decreased as the coating layer thickness increased, with the UHA/PS10-filled DRCs group showing the lowest effect. This could mainly be attributed to the reduced dissolution rate of thicker PS layers, leading to less calcium and phosphorus ion release. Nevertheless, all UHA/PSx groups still exhibited superior mineralization compared with the blank control, confirming the beneficial role of UHA in supporting odontogenic differentiation. The PS coating thickness affects the bioactivity of DRCs, thus a more systematic study on the influence of PS thickness on the comprehensive performance of DRCs should be studied in future work, finally verifying the optimized value of the PS shell thickness.

## 4. Conclusions

In summary, a novel structure–functional-integrated particle (UHA/PSx) was successfully fabricated by coating porous silica onto UHA surfaces via a controllable process, and the reaction time of the porous silica coating had a significant effect on the morphologies of UHA/PSx. When used as filler in DRCs, UHA/PSx showed a dual physical micromechanical interlocking structure when mixing with resin matrix, particularly UHA/PS5 with the optimize structure and the appropriate layer thickness. Among all composites, UHA/PS5-filled DRCs presented the best filler/resin matrix interface interaction and excellent mechanical properties, demonstrating the effectiveness of the surface modification developed here. Additionally, the coating porous silica layer on UHA surface effectively reduces the water absorption and solubility of corresponding filled DRCs. Based on the cytological properties of various DRCs, UHA/PSx-filled DRCs exhibited bioactivity, enhancing the mineralized differentiation of hDPSCs. Therefore, this surface modification method could be considered as a prospective method to fabricate DRCs with strong interfacial adhesion, superior mechanical performance, and biological benefits. The developed UHA/PSx filler system shows great potential for clinical use in restorative dentistry, and the future efforts should focus on scaling up production, fulfilling clinical regulatory requirements, and validating the material’s performance in in vivo settings.

## Figures and Tables

**Table 1:** Textural characteristics of UHA/PSx particles.

Particle	Specific Surface Area, S_BET_ (m^2^/g) ^a^	Cumulative Pore Volume, V_total_ (cm^3^/g) ^b^
UHA	28.27	0.15
UHA/PS3	35.13	0.18
UHA/PS5	39.16	0.19
UHA/PS7	41.37	0.22
UHA/PS10	59.72	0.24

^a^ S_BET_ is the specific surface area measured from N_2_ physisorption. ^b^ V_total_ is the cumulative pore volume measured at P/P_0_ = 0.99.

**Table 2:** The content of Si, Ca, P and C in UHA and UHA/PSx evaluated by XPS and ICP-AES.

Method		Sample	UHA	UHA/PS3	UHA/PS5	UHA/PS7	UHA/PS10
	Element						
XPS (Ar%)	Si		0.70	5.06	8.74	14.43	15.98
	Ca		17.24	14.45	11.51	6.10	5.47
	O		57.71	54.51	52.55	46.14	48.69
	P		13.42	11.48	9.26	4.85	4.71
	C		10.92	14.50	18.24	28.48	25.13
ICP-AES (mg/g)	Si ^a^		/	2.40	4.67	13.55	28.93

^a^ Based on the quantitative measurement and analysis of ICP-AES.

## Data Availability

The original contributions presented in this study are included in the article/Supplementary Materials. Further inquiries can be directed to the corresponding authors.

## Supplementary Materials

The following supporting information can be downloaded at: https://www.mdpi.com/article/10.3390/polym17172384/s1.
