# Supplementary material for: Dual Micromechanical Interlocking Through Filler Surface Modification for Enhanced Dental Composites
Source: Polymers (Basel). 2025 Aug 31;17(17):2384. doi: 10.3390/polym17172384 (PMC12431051; doi:10.3390/polym17172384)
Supplement: Supplementary file 1 [file polymers-17-02384-s001.zip › polymers-3814008-supplementary.pdf]

Supporting Information:

Table S1. List of primers for RT-PCR in this study.

	Forward	Reverse
**ALP**	AACATCAGGGACATTGACGTG	GTATCTCGGTTTGAAGCTCTTCC
**OPN**	CTCCATTGACTCGAACGACTC	CAGGTCTGCGAAACTTCTTAGAT
**OSX**	GAGGCAACTGGCTAGGTGG	CTGGATTAAGGGGAGCAAAGTC
**RUNX2**	CCGCCTCAGTGATTTAGGGC	GGGTCTGTAATCTGACTCTGTCC
**DMP1**	TCTTTGTGAACTACGGAGGGTA	CACTGCTCTCCAAGGGTGG
**DSPP**	GCTGGCCTGGATAATTCCGA	CTCCTGGCCCTTGCTGTTAT
**GADPH**	GGAGCGAGATCCCTCCAAAAT	GGCTGTTGTCATACTTCTCATGG

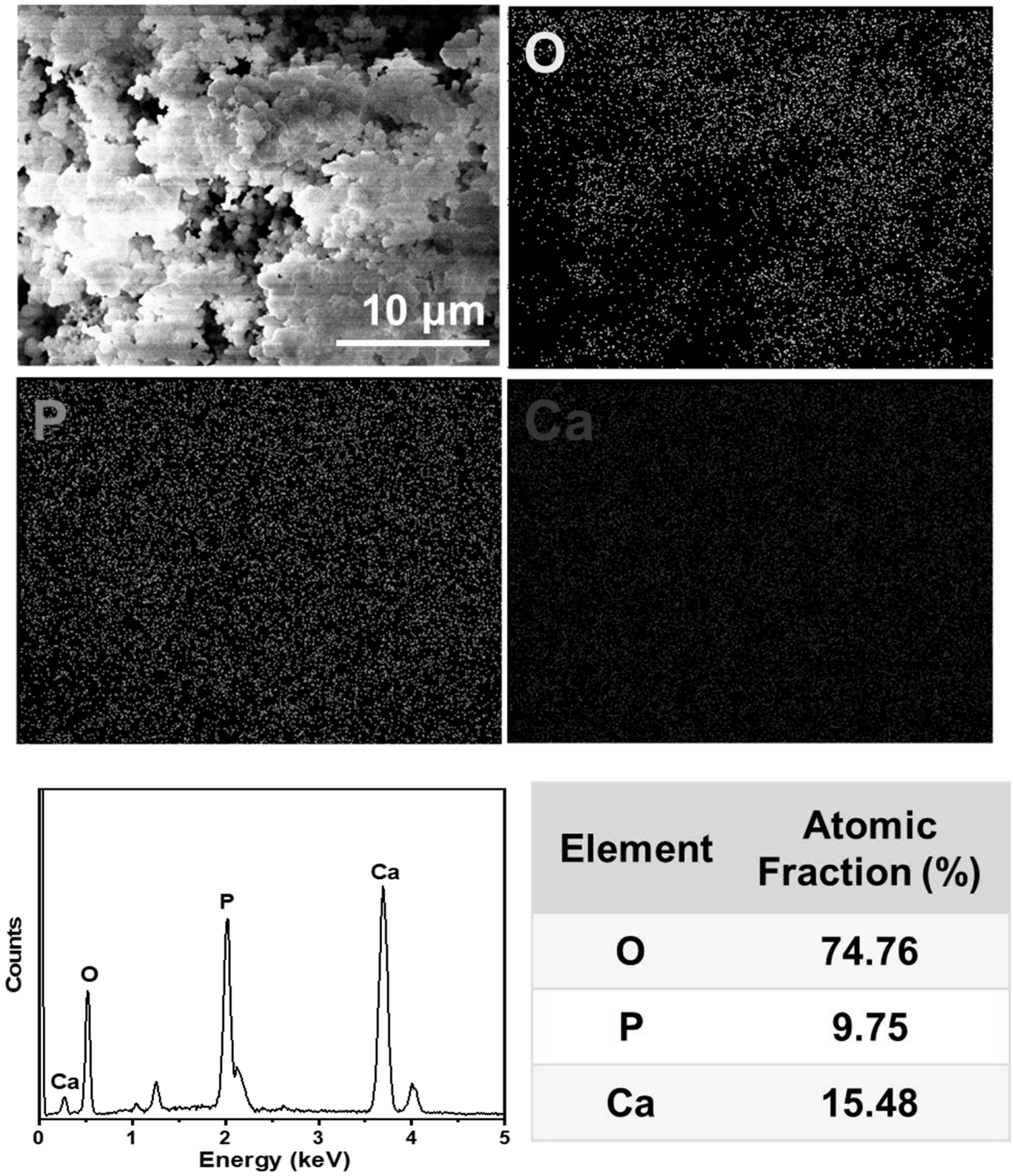

Element	Atomic Fraction (%)
O	74.76
P	9.75
Ca	15.48

Figure S1. SEM images, element mapping, and EDS analysis of the representative UHA/PS5-filled dental resin composite after soaking in SBF for 15 days.
